# The survival time of the ventriculo-peritoneal-shunt in children with hydrocephalus is dependent on the type of valve implanted

**DOI:** 10.1007/s00383-023-05395-0

**Published:** 2023-02-13

**Authors:** Danielle S. Wendling-Keim, Elena Kren, Oliver Muensterer, Markus Lehner

**Affiliations:** 1 Department of Pediatric Surgery, Dr. von Hauner Children’s Hospital, Ludwig-Maximilians-University, Munich, Germany; 2 Department of Pediatric Surgery, Children’s Hospital, Luzerner Kantonsspital, Lucerne, Switzerland

**Keywords:** Ventriculo-Peritoneal Shunt, Hydrocephalus, Gravitational valve, Differential pressure valve

## Abstract

**Purpose:**

Despite constantly improving developments in ventriculo-peritoneal shunt systems, most patients with hydrocephalus require revision or replacement at some point of time. Therefore, this study aimed to analyse parameters that are associated with shunt dysfunction.

**Methods:**

In this retrospective study, we included 81 patients aged 0–17 who were treated at our institution. Demographic data, etiology of the hydrocephalus, type of valve implanted, reason for any revision procedures, any complications and survival time of the ventriculo-peritoneal shunts were detected. Statistical analysis was performed using SPSS. The significance level was set at *p* ≤ 0.05.

**Results:**

Over a mean study period of 18 years, we analyzed 226 valves subjected to 146 revision operations in 81 patients. The etiology of the hydrocephalus (*p* = 0.874) and the age of the child at the time of VP shunt implantation (*p* = 0.308) did not have any impact on the shunt survival time. However, the type of the valve significantly changed the survival time of the shunt (*p* = 0.030). Pressure differential valves presented a longer survival time than gravitational valves.

**Conclusion:**

The majority of patients in this study needed at least one replacement of the initial shunt system. Pressure differential valves may be beneficial for the survival time of the shunt system.

## Introduction

The standard treatment for hydrocephalus is the implantation of a ventriculo-peritoneal shunt (VP shunt) [1]. Nevertheless, VP shunt revisions due to shunt dysfunction, disconnection, obstruction, or infection are common complication, especially in pediatric patients [2, 3]. Antibacterial impregnation as well as the position of the valve, the etiology, the surgeon experience, and the age of the child have been under investigation as well as the type of the valve (differential, gravitational, programmable, non-programmable) [4, 5]. Nevertheless, to date factors with an impact on the VP shunt survival time are under investigation. No prognostic parameters for VP shunt failure have been discovered and measures to prevent revision procedures are controversial [6, 7].

Therefore, it is the goal of our study to identify parameters that influence the risk of shunt failure leading to revision procedures to minimize shunt failure rates and the need for revision surgery.

## Patients and methods

In a retrospective monocentric study data of 117 patients aged 0–18 years who presented with hydrocephalus to our tertiary care hospital were assessed and analyzed. According to the inclusion criteria all patients aged 0–18 years presenting to our institution with hydrocephalus who were treated with a ventriculo-peritoneal shunt entered the study. After the exclusion of patients with bilateral VP shunts, with missing data regarding the primary surgery or dropout during the follow-up period, 81 patients and 226 VP shunts were analyzed. First, data from the primary procedure were studied; for the investigation of the revision surgery, every valve was handled as a separate case. Preterm infants with a gestational age of less than 37 weeks at birth were evaluated within the study population as well as separately. Further, patients were grouped into age blocks according to the age at primary shunt implantation, namely younger than 30 days, 30–59 days, and 60–100 days and older than 100 days.

Shunt survival time as the primary outcome was defined as the time from shunt implantation to the occurrence of any VP shunt-related complication leading to revision surgery. Complications leading to revision surgery included obstruction, disconnection, infection, and dysfunction (over- or underdrainage). Time of revision surgery was the start point of the next survival time. For the separate calculation of the survival times of the valves only. In these cases, the valve was considered as survived if the cause of shunt failure was disconnection or obstruction of the catheter or infection. The mean observation time was 226 months with a minimum of 108 months and a maximum of 441 months.

All data were irreversibly anonymized. Data were expressed as means ± standard deviation and subjected to Student’s unpaired *t*-test and Spearman’s rank correlation. A level of *p* < 0.05 was considered significant.

## Results

### Patient demographics

In this study, 81 patients, 43 male patients and 38 female patients respectively, were included. During the course of the study and due to revision surgery, 226 shunts were observed in these 81 patients. Here, the hydrocephalus was due to intracerebral hemorrhage (ICH) in 49.4% of cases (*n* = 40), associated with meningomyelocele (MMC) in 23.5% of cases (*n* = 19) and congenital in 19.8% (*n* = 16) of cases. In 3.7% (*n* = 3) the hydrocephalus occurred after an infection, in 1 child (1.2%) after traumatic brain injury (TBI) and in 2.5% (*n* = 2) the etiology was unknown. Of the aforementioned 81 patients, 35 children were preterm infants, and within this subgroup the majority of 82.9% (*n* = 29) had post-hemorrhagic hydrocephalus, 5.7% (*n* = 2) had a congenital hydrocephalus, in 8.6% (*n* = 3) the hydrocephalus was associated with meningomyelocele and in 2.9% (*n* = 1) the etiology was unknown. Accordingly, and as expected, the etiology of the hydrocephalus was significantly different (*p* < 0.001) in maturely and immaturely born babies as ICH being the main cause of hydrocephalus in this group. A synopsis of the baseline characteristics of the patients included in this study is assembled in Table 1.

**Table 1:** Baseline table with the characteristics of the patients, valves and complications from this study

Primary VP shunt	PaediGAV		PS medical		Pro medics Delta		Various		Total	
	*n*	%	*n*	%	*n*	%	*n*	%	*n*	%
Total	32	39.5	23	28.4	5	6.2	21	25.9	81	100.0
Male patients	13	40.6	14	60.9	5	100.0	11	52.4	43	53.1
Preterm patients	14	43.8	11	47.8	2	40.0	8	38.1	35	43.2
Age at implantation										
Up to 30 days	15	46.9	12	52.2	1	20.0	10	47.6	38	46.9
Up to 60 days	4	12.5	2	8.7	3	60.0	4	19.0	13	16.0
Up to 100 days	4	12.5	3	13.0	1	20.0	2	9.5	10	12.3
More than 100 days	9	28.1	6	26.1	0	0	5	23.8	20	24.7
Etiology										
Post-hemorrhagic	18	56.3	11	47.8	3	60.0	8	38.1	40	49.4
Congenital	4	12.5	5	21.7	1	20.0	6	28.6	16	19.8
Post infectious	1	3.1	1	4.3	0	0	1	4.8	3	3.7
MMC	7	21.9	6	26.1	1	20.0	5	23.8	19	23.5
Traumatic	0	0	0	0	0	0	1	4.8	1	1.2
Unknown	2	6.3	0	0	0	0	0	0	2	2.5
Cause of shunt failure										
Valve-dysfunction	9	39.1	4	23.5	2	66.7	8	50.0	23	39.0
Infection	4	17.4	0	0	0	0	1	6.3	5	8.5
Central disconnection	2	8.7	6	35.3	0	0	2	12.5	10	16.9
Peripheral disconnection	4	17.4	2	11.8	1	33.3	4	25.0	11	18.6
Obstruction	4	17.4	5	29.4	0	0	1	6.3	10	16.9

All Valves included in the study	PaediGAV		ProGAV		PS medical		Pro medics delta		Various		Total	
	*n*	%	*n*	%	*n*	%	*n*	%	*n*	%	*n*	%
Total	81	35.8	44	19.5	47	20.8	10	4.4	44	19.5	226	100.0
Male	36	44.4	29	65.9	29	61.7	10	100.0	20	45.5	124	54.9
Preterm	40	49.4	22	50.0	21	44.7	3	30.0	16	36.4	102	45.1
Etiology												
Post-hemorrhagic	45	55.6	20	45.5	22	46.8	5	50.0	15	34.1	107	47.3
Congenital	14	17.3	9	20.5	13	27.7	3	30.0	16	36.4	55	24.3
Post infectious	2	2.5	3	6.8	1	2.1	0	0	2	4.5	8	3.5
MMC	16	19.8	12	27.3	11	23.4	2	20.0	9	20.5	50	22.1
Traumatic	1	1.2	0	0	0	0	0	0	2	4.5	3	1.3
Unknown	3	3.7	0	0	0	0	0	0	0	0	3	1.3
Cause of shunt failure												
Valve-dysfunction	22	41.5	7	38.9	11	33.3	3	60.0	18	48.6	61	41.8
Infection	5	9.4	0	0	0	0	0	0	1	2.7	6	4.1
Central disconnection	2	3.8	2	11.1	6	18.2	0	0	3	8.1	13	8.9
Peripheral disconnection	11	20.8	4	22.2	8	24.2	2	40.0	11	29.7	36	24.7
Obstruction	13	24.5	5	27.8	8	24.2	0	0	4	10.4	30	20.5

### The etiology of the hydrocephalus and age at implantation does not change shunt survival

**Fig. 1:**
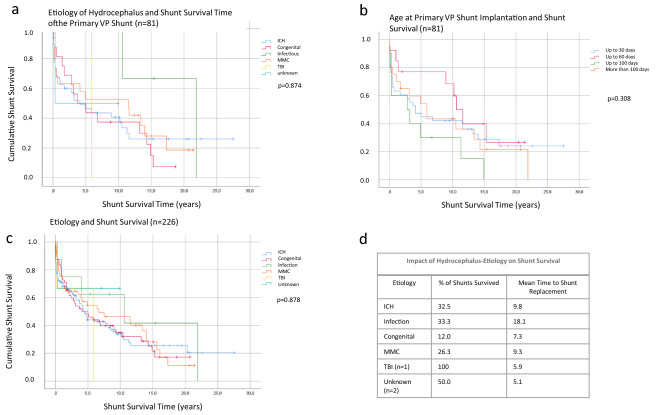
Etiology of the hydrocephalus and age at implantation. The etiology of the hydrocephalus and the age of the patients does not change the survival time of the vp shunt. The survival curves are shown for the primary vp shunts (**a**) as well as for all vp shunts implanted (**c**). **b** depicts the independence of the survival time of the age at implantation while details of shunt survival are given in **d**)

Shunt survival of the initial VP shunt system was explored using Kaplan–Meier-Curves. Investigation of the interrelation of the etiology of the hydrocephalus and the shunt survival revealed that the shunt survival time was not significantly changed by the cause of the hydrocephalus (*p* = 0.874; Fig. 1a). To investigate the influence of the age of the patient at implantation we grouped the patients into age clusters as described in the methods part and found that the age at implantation did not significantly (*p* = 0.308) alter the shunt survival of the primary shunt system (Fig. 1b). In a next step, we captured 146 revisional operations that were carried out in 59 of the 81 patients. This led to a total of 226 valves that were analyzed. As shown in Table 1, the indications for the revision procedures mainly resulted from a dysfunction of the valve or a disconnection and rarely from an infection. As we investigated all valves including the revised ones we found a similar result as after primary implantation of the VP shunt system: The etiology of the hydrocephalus had no impact (*p* = 0.878) on the shunt survival (Fig. 1c and d).

### The type of the valve implanted changes VP shunt survival

**Fig. 2:**
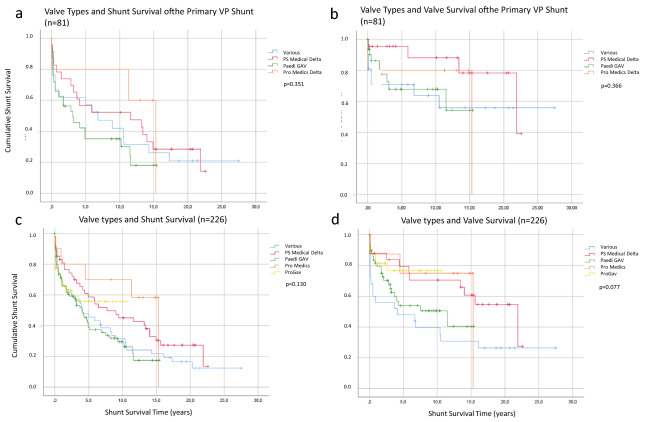
Shunt Survival Curves and type of valve. The survival curves of the various valves implanted primarily (**a**, **b**) and the survival time of all valves under investigation (**c**, **d**) are demonstrated here. Further, the curves differentiate between shunt survival (**a**, **c**) and valve survival (**b**, **d**)

The next parameter under investigation was the type of valve. In our study, the most frequently used valves for the primary VP shunt implantation were gravitational and differential pressure valves (Table 1). The type of valve was chosen according to the preference of the responsible surgeon. The proGAV system was not available during the period of the primary implantation yet. In the first step we found that the various types of valves implanted during the primary surgery did not change the survival of the complete shunt system (Fig. 2a; *p* = 0.351). To specifically differentiate if the shunt failure was due to the valve’s dysfunction (over- or underdrainage or obstruction), we considered the valve as “survived” if the cause of shunt failure clearly was found in the catheter (obstruction or disconnection) or due to an infection. Considering this, the various valve types from the primary operation did not reveal different valve survival times in this study (*p* = 0.366; Fig. 2b). This finding was confirmed by the evaluation of the shunt survival of all complete shunt systems implanted including the revised systems (Fig. 2c). However, when we explored the valves only of all 226 shunts of this study the result was not quite significant, but we found a tendency that the type of the valve may make a difference (*p* = 0.077) (Fig. 2d).

**Fig. 3:**
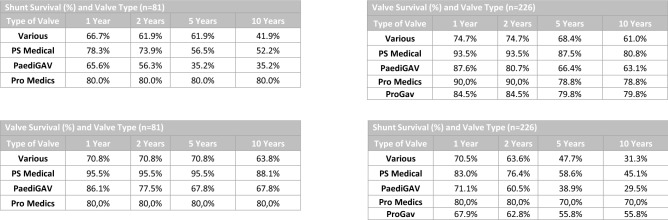
VP Shunt survival time and Valve survival time**.** The details of shunt survival and valve survival of the primary shunt systems and all shunts under investigation are shown here

The detailed results of the survival times are shown in Fig. 3 and corroborate the Kaplan–Meier curves.

**Fig. 4:**
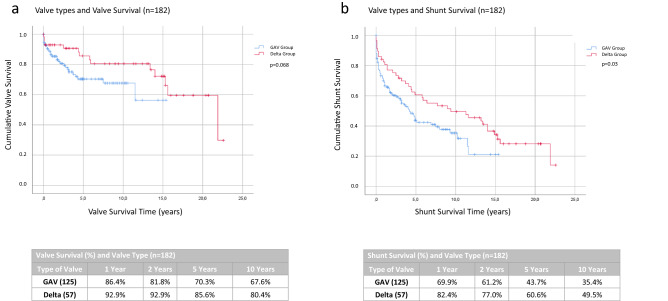
The mechanism of the valve type changes the survival time of the shunt system. The valve survival time of the differential pressure valves shows a tendency to be longer than the gravitational valves (**a**) and the survival time of the complete shunt system is significantly longer in differential pressure valves than in gravitational valves (**b**)

However, as we focused on the mechanism of the valves and grouped them into valves with a gravitational unit (GAV group: PaediGAV and ProGAV) and pressure differential valves with a siphon control device only (Delta group: PS Medical Delta and Pro Medics Delta) and by omitting the other valves (*n* = 182) we found a slight difference. The mean valve survival time was 10.7 years in the gravitational group and 16.9 years in the differential pressure group, this difference was close to significant (*p* = 0.068; Fig. 4a). Further, the survival curves of the complete shunt systems indicated a significantly longer shunt survival time in the differential pressure group than in the gravitational group with a *p*-value of 0.030 (see Figure 4b).

The next point of interest was whether the adjustability of the ProGAV valves could improve the survival time of the shunt. We grouped 138 non-adjustable valves and 44 adjustable valves and found that the comparison of the groups did not indicate any significant difference (*p* = 0.830) so the adjustability did not improve the outcome in this study.

### Complications leading to VP shunt replacement

**Fig. 5:**
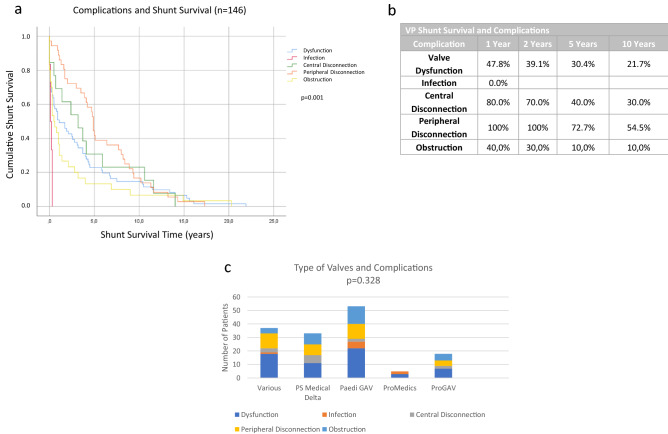
The complications leading to revision surgery. The complication causing the earliest shunt failure was an infection in patients in this study (**a**, **b**). However, the complications leading to the replacement of the vp shunt were independent of the type of valve (**c**)

To identify reasons for VP shunt failure that led to the replacement of parts of the system or of the complete shunt we created Kaplan–Meier-Curves showing the shunt survival depending on the causes of VP shunt failure which were valve dysfunction, infection, disconnection and obstruction (Table 1). The most common reason for VP shunt failure was valve dysfunction and disconnection of the catheter. The rare reasons, namely infection and obstruction, occurred significantly (*p* < 0.001) earlier after implantation of the VP shunt than valve dysfunction or disconnection (Fig. 5a and b). Nevertheless, the type of the valve did not affect the complications that occurred (Fig. 5c).

## Discussion

This study demonstrates that the selection of the type of valve of the VP shunt system used to treat hydrocephalus does not significantly influence the survival time of the valve. This was the case for the primary VP shunt as well as the VP shunts that were implanted during revisional surgery. To specifically differentiate the impact of the valve, we regarded valves as intact if the catheter of the VP shunt was replaced only. Notably, the survival time of the Delta valves was not significantly longer compared to the GAV valves when we focused on the valves whereas it was longer when the complete shunt survival was calculated. This possibly means that the shunt survival of the Delta system was longer in this study group, but not due to the mechanism of the valves but rather a better connection of the catheter. Certainly, our study reveals that the GAV group was not superior to the Delta group. And it is even more notable that the adjustable valves did not yield any benefit compared to non-adjustable valves. The high VP shunt failure rate especially in children remains a known problem [2, 8, 9]. With this study, we show that the type of valve may play a role for shunt survival and needs further prospective studies with a new focus on adjustable valves.

Although previous studies have suggested that the type of valve does not matter [4], these results came from a study with a short follow up period whereas the observation period of our study was exceptionally long with a minimum of 9 years follow-up time. Especially VP shunt failure due to dysfunction of the valve (over- or underdrainage) is known to occur late during the course after shunt placement.

A further explanation for the unexpected non-superiority of the GAV group compared to the Delta group are observations published by Pollack [10] (1999) and later Eymann [11] (2007) that the shunt system survival is dependent on the timing of surgery. Both showed a reduced survival time of shunts inserted at revision compared to those implanted at primary surgery [10, 12]. All ProGAV valves observed were implanted only during revisional surgery, and not as a primary valve. The long observation period of 10 years showed improved survival of paediGAV used at primary surgery (35.2%) compared with those used at revision surgery (29.5%). To better focus on the mechanism of the valves, they were divided into groups with delta unit or with the gravitationally controlled unit. Shunt survival times were significantly different in the overall collective as well as in the preterm group. Delta-unit valves showed 1- and 2-year shunt survival of 82.4% and 77.0%, respectively, in the overall collective and 83.3% and 83.3%, respectively, in the preterm infants. For gravitationally controlled valves, this was only 69.9% and 61.2% in the overall collective and 67.5% and 60.5% in the preterm infants, respectively. However, it must be considered here that the gravity-controlled valves were developed later, and therefore not only the observation period is shorter but also the shunt survival time. In addition, a large proportion of these valves were used in revision surgeries, which also minimizes survival. It also showed that in the delta group, 66.7% of the shunt systems had to be revised. In the gravitationally controlled valve group, the percentage was only 62.9%.

The last distinction between the valve types was the possibility of adjustability, especially regarding recurrent over- and underdrainage. Here, there was no significant difference in shunt and valve survival both in the overall collective and in preterm infants. However, the significant graphical difference should not be ignored. Here, a clear superiority of the adjustable proGAV was shown. The 5-year shunt survival was 55.8% in the total collective and as high as 62.0% in the preterm infants. In terms of valve survival, the values were even better. Thus, valve survival for the proGAV after 5 years was 79.8% for the total collective and even 82.6% for the preterm infants. This compares with a 5-year shunt survival of 48.5% and a valve survival of 75.1% for the nonadjustable valves and a shunt survival of 38.4% and a valve survival of 64.5% for the preterm infants. As mentioned above for the gravity-guided valves, the shorter observation period due to the new technology is also a possible reason for the statistically poor performance. In terms of absolute numbers, it could be clearly shown that the proGAV required significantly fewer revisions. In the total collective, 59.1% and in the preterm infants even 63.6% did not require a revision. In contrast, only about one-third of the non-adjustable valves required no revision. This final evaluation makes it clear that especially the gravitationally controlled valves and here in particular the adjustable forms need further investigations to show a possible superiority with first implantation and a similarly long observation period as with the older valve types. Our study only suggests this at the current time. Such comparisons are not described in the literature to date.

Further, for prognosis and counseling, our results that the etiology of shunt implantation does not change the shunt failure rate yield important information for the affected patients and their families. Shannon (2012) [13] showed that etiology did not influence shunt survival [13]. Also, our analyses using Kaplan–Meier curves, demonstrate no significant differences in shunt survival depending on the etiology of hydrocephalus after either primary or revision surgeries. In contrast, Simon postulated in 2012 that ICH is associated with an increased risk of subsequent shunt surgery within 12 months of shunt implantation [14]. This finding came from a comparison of posthemorrhagic hydrocephalus with hydrocephalus in aqueduct stenosis.

Also, in contrast to other studies under review [15], we did not find any correlation between shunt failure and age at implantation, and infections were rare in the patients we investigated. Age at implantation is another parameter discussed in the literature. Young patient age at shunt placement has been repeatedly postulated as a risk factor for revisions [6, 8, 13, 15, 16]. This was not a relevant factor in our study parameter since age did not have any impact on the rate of shunt failure in this study (*p* > 0.05).

Limitations of the study are the retrospective design. The selection of the type of valve was therefore not random or systematic but depended on the preference of the operating surgeon. Further, we did not return defect valves to the manufacturer for analysis. This is possible with some companies and may yield further information on how to avoid dysfunctions. Other parameters, for example, the protein content of the liquor, was not investigated. This could be a further parameter influencing the occurrence of obstruction complications. In addition, the introduction of the adjustable ProGAV valve as a new method has led to smaller numbers in this study and a smaller observation period compared to the Delta valves. Future studies should focus on these new valves to reevaluate the adjustable valves within a larger and prospective study.

## Conclusion

In this study, we demonstrated that the selection of the valve may play an important role in the VP shunt survival time in children with hydrocephalus. Although we could not show that VP shunts with GAV valves may yield a benefit for the shunt survival time and the statistics revealed a possible superiority of the Delta valves, our results suggest that this outcome may be due to the long observation period of this study and the GAV valves being newly introduced during the study period. For counseling of the affected patients and families our results suggest that the etiology of the hydrocephalus as well as the age at implantation of the VP shunt does not change the need for revisional surgery.

## Data Availability

Datasets analyzed during the current study are available upon request emailed to the corresponding author.

## References

[1] Tully HM, Dobyns WB (2014). Infantile hydrocephalus: a review of epidemiology, classification and causes. Eur J Med Genet.

[2] Merkler AE, Ch’ang J, Parker WE, Murthy SB, Kamel H (2017). The rate of complications after ventriculoperitoneal shunt surgery. World Neurosurg.

[3] Lim J, Tang AR, Liles C, Hysong AA, Hale AT, Bonfield CM, Naftel RP, Wellons JC, Shannon CN (2019). The cost of hydrocephalus: a cost-effectiveness model for evaluating surgical techniques. J Neurosurg Pediatr.

[4] Haberl EJ, Messing-Juenger M, Schuhmann M, Eymann R, Cedzich C, Fritsch MJ, Kiefer M, van Lindert EJ, Geyer C, Lehner M, Rohde V, Stroux A, von Berenberg P (2009). Experiences with a gravity-assisted valve in hydrocephalic children: clinical article. J Neurosurg Pediatr.

[5] Hall BJ, Gillespie CS, Sunderland GJ, Conroy EJ, Hennigan D, Jenkinson MD, Pettorini B, Mallucci C (2021). Infant hydrocephalus: what valve first?. Child’s Nerv Syst.

[6] Venable GT, Rossi NB, Jones GM, Khan NR, Smalley ZS, Roberts ML, Klimo P (2016). The preventable shunt revision rate: a potential quality metric for pediatric shunt surgery. J Neurosurg Pediatr.

[7] Dave P, Venable GT, Jones TL, Khan NR, Albert GW, Chern JJ, Wheelus JL, Governale LS, Huntoon KM, Maher CO, Bruzek AK, Mangano FT, Mehta V, Beaudoin W, Naftel RP, Basem J, Whitney A, Shimony N, Rodriguez LF, Vaughn BN, Klimo P (2019). The preventable shunt revision rate: a multicenter evaluation. Clin Neurosurg.

[8] Riva-Cambrin J, Kestle JRW, Holubkov R, Butler J, Kulkarni Av, Drake J, Whitehead WE, Wellons JC, Shannon CN, Tamber MS, Limbrick DD, Rozzelle C, Browd SR, Simon TD (2016). Risk factors for shunt malfunction in pediatric hydrocephalus: a multicenter prospective cohort study. J Neurosurg Pediatr.

[9] Reddy GK, Bollam P, Caldito G (2014). Long-term outcomes of ventriculoperitoneal shunt surgery in patients with hydrocephalus. World Neurosurg.

[10] Pollack IF, Albright AL, Adelson PD (1999). A randomized, controlled study of a programmable shunt valve versus a conventional valve for patients with hydrocephalus. Neurosurgery.

[11] Eymann R, Steudel WI, Kiefer M (2007). Pediatric gravitational shunts: initial results from a prospective study. J Neurosurg.

[12] Kestle JRW, Riva-Cambrin J (2019). Prospective multicenter studies in pediatric hydrocephalus. J Neurosurg Pediatr.

[13] Shannon CN, Acakpo-Satchivi L, Kirby RS, Franklin FA, Wellons JC (2012). Ventriculoperitoneal shunt failure: an institutional review of 2-year survival rates. Child’s Nerv Syst.

[14] Simon TD, Whitlock KB, Riva-Cambrin J, Kestle JRW, Rosenfeld M, Dean JM, Holubkov R, Langley M, Hamblett NM (2012). Revision surgeries are associated with significant increased risk of subsequent cerebrospinal fluid shunt infection. Pediatr Infect Dis J.

[15] Paff M, Alexandru-Abrams D, Muhonen M, Loudon W (2018). Ventriculoperitoneal shunt complications: a review. Interdiscip Neurosurg.

[16] Ved R, Bentley E, Amato-Watkins A, Lang J, Zilani G, Bhatti I, Leach P (2019). One year failure rates for de-novo ventriculo-peritoneal shunts in under 3-month-old children. Br J Neurosurg.

